# Myonectin inhibits the differentiation of osteoblasts and osteoclasts in mouse cells

**DOI:** 10.1016/j.heliyon.2020.e03967

**Published:** 2020-05-15

**Authors:** Miku Kawaguchi, Naoyuki Kawao, Yoshimasa Takafuji, Masayoshi Ishida, Hiroshi Kaji

**Affiliations:** Department of Physiology and Regenerative Medicine, Kindai University Faculty of Medicine, 377-2 Ohnohigashi, Osakasayama, Osaka, 589-8511, Japan

**Keywords:** Cell biology, Physiology, Bone, Endocrinology, Metabolism, Musculoskeletal system, Myonectin, Osteoblast, Osteoclast, Myokine, Mitochondria

## Abstract

Myonectin is a myokine, which is involved in the pathophysiology of diabetes and obesity, and various myokines are involved in the interactions between skeletal muscle and bone. However, roles of myonectin in bone have still remained unknown. We therefore examined the effects of myonectin on mouse osteoblast and osteoclast differentiation *in vitro*. Myonectin significantly suppressed the mRNA levels of osteogenic genes and alkaline phosphatase (ALP) activity in mouse osteoblasts. As for osteoclasts, myonectin significantly suppressed osteoclast formation as well as the mRNA levels of osteoclast-related genes enhanced by receptor activator nuclear factor κB ligand (RANKL) from mouse monocytic RAW264.7 cells. Moreover, myonectin significantly suppressed osteoclast formation from mouse bone marrow cells in the presence of macrophage-colony stimulating factor and RANKL. On the other hand, myonectin significantly suppressed RANKL-induced oxygen consumption rate and peroxisome proliferator-activated receptor γ coactivator-1β mRNA levels in RAW264.7 cells, although myonectin did not affect these mitochondrial biogenesis parameters in mouse osteoblasts. In conclusion, the present study demonstrated that myonectin suppresses the differentiation and ALP activity in mouse osteoblasts. Moreover, myonectin suppressed osteoclast differentiation from mouse bone marrow and RAW264.7 cells partly through an inhibition of mitochondrial biogenesis.

## 1 Introduction

Myokines are factors secreted from the skeletal muscles, and many myokines play some roles in metabolic regulation through the circulation during physical exercise. Myonectin, also called C1q (complement component 1q)/tumor necrosis factor-related protein 15 (CTRP15)/erythroferrone is a myokine, which is predominantly expressed in skeletal muscle tissues [1]. Its domain structure is homologous to well-known adipocytokine, adiponectin [1]. Acute exercise and nutrients, such as glucose and fatty acid, enhance the expression of myonectin as the main regulators [2, 3, 4], which promotes fatty acid uptake in adipocytes and hepatocytes [3], although the contradictory data was reported about the effects of exercise on muscle myonectin expression [5]. Myonectin expression is predominant in slow-twitch muscles, compared to that in fast-twitch muscles [2]. Numerous studies suggest that myonectin is involved in the abnormalities of glucose, lipid and energy metabolism, such as diabetes and obesity [4, 6, 7, 8, 9]. On the other hand, myonectin induced in erythroblasts links stress erythropoiesis to iron mobilization in liver in response to blood loss in mice [10], and Otaka et al. recently reported that myonectin improves myocardiac injury through a reduction in cardiac myocyte apoptosis and macrophage-related inflammation in mice [11]. These findings suggest that myonectin is an important myokine for the regulation of metabolic state. However, roles of myonectin in bone have still remained unknown.

The clinical relationships between sarcopenia and osteoporosis raised the increasing research interests in the interactions between skeletal muscles and bone [12]. Myokines have been noted as crucial mediators for the muscle/bone relationships, which may be a target for drug development of both sarcopenia and osteoporosis [12]. Since myonectin, irisin and fibroblast growth factor-21 have been known as myokines affecting glucose and energy metabolism, numerous studies suggest that irisin is involved in the pathophysiology of osteoporosis as a myokine linking muscle to bone [12]. Based on these findings, we speculated that myonectin might be related to the muscle/bone relationships as a myokine linking muscle to bone.

The present study was therefore performed to investigate the effects of myonectin on mouse bone cells, including osteoblasts and osteoclasts.

## 2 Materials and methods

### 2.1 Materials

Recombinant myonectin was purchased from Aviscera Bioscience (Santa Clara, CA, USA). Recombinant receptor activator nuclear factor κB ligand (RANKL) and macrophage colony-stimulating factor (M-CSF) were obtained from Wako (Osaka, Japan).

### 2.2 Cell culture

Mouse monocytic RAW264.7 cells were obtained from ATCC (Manassas, VA, USA) and cultured in Dulbecco's Modified Eagle's Medium (DMEM; Wako) with 10% FBS and 1% penicillin/streptomycin. Medium was changed twice a week.

### 2.3 Animals

C57BL/6J mice were obtained from CLEA Japan (Tokyo, Japan). Animal experiments were performed according to the guidelines of the National Institutes of Health and the institutional rules for the use and care of laboratory animals at Kindai University. All animal experiments were approved by the Experimental Animal Welfare Committee of Kindai University (Permit number: KAME-27-029).

### 2.4 Preparation of primary osteoblasts

Primary osteoblasts were collected from the calvaria of new born C57BL/6J mice, as described previously [13]. Briefly, after the mice were euthanized with excess isoflurane, the calvaria was removed and digested with dissociation medium [Minimum Essential Medium Alpha Modification (αMEM; Wako) with 1 mg/mL collagenase and 0.25% trypsin] for 20 min at 37 °C. Osteoblasts were grown in αMEM with 10% FBS and 1% penicillin/streptomycin. The character of primary osteoblasts was confirmed as alkaline phosphatase (ALP)-positive cells.

### 2.5 Real-time polymerase chain reaction (PCR)

Total RNA extraction and real-time PCR were performed, as previously described [14]. Total RNA was isolated from cells using an RNeasy Mini Kit (Qiagen, Hilden, Germany). A High-capacity cDNA Reverse Transcription Kit (Applied Biosystems, Foster, CA, USA) was used for reverse transcription reaction. Real-time PCR was performed using a StepOnePlus real time PCR systems (Applied Biosystems) with a Fast SYBR Green Master mix (Applied Biosystems). The specific mRNA amplification of the target was determined as the Ct value followed by normalization with the glyceraldehyde-3-phosphate dehydrogenase (GAPDH) level. Primer sequences were shown in Table S1.

### 2.6 ALP activity

ALP activity in primary osteoblasts was analyzed, as described previously [15]. Primary osteoblasts were cultured in 24-well plate until reaching confluent. Osteoblasts were, then, washed 3 times with PBS, and distilled water (200 μl/well) was added to each well. ALP activity was analyzed using Lab assay ALP kit (Wako), according to the manufacturer's instructions. The absorbance was measured at 405 nm using a microplate reader. Total protein concentration was determined by Protein Assay BCA Kit (Pierce, Rockford, IL, USA) and ALP activity was defined as [unit/protein (μg)].

### 2.7 Mineralization

Mineralization of calvarial osteoblasts was determined by Alizarin Red staining, as described previously [13]. After reaching confluence, osteoblasts were cultured in αMEM with 10% FBS, 10 mM β-glycerophosphate and 1% penicillin/streptomycin for 3 weeks. The cells were fixed with ice-cold 70% ethanol and stained with Alizarin Red S solution. For quantification, the stained cells were destained with 10% cetylpyridinium chloride, after which the extracted stain was transferred to a 96-well plate and the absorbance was measured at 570 nm.

### 2.8 Osteoclast formation

Osteoclast formation was induced in mouse bone marrow cells and RAW264.7 cells, as described previously [14, 16]. Briefly, bone marrow cells were collected from the femur and tibia of C57BL/6J mice, cultured in αMEM with 10% FBS, and 50 ng/ml M-CSF for 3 days. Then, osteoclasts were formed in αMEM with 10% FBS, 50 ng/ml M-CSF and 75 ng/ml RANKL for further 4 days. RAW264.7 cells were cultured in αMEM with 10% FBS and 75 ng/ml RANKL for 4 days to induce osteoclast formation. Detection of osteoclasts was performed using a tartrate-resistant acid phosphatase (TRAP) staining kit (Wako), and the numbers of TRAP-positive multinucleated cells (MNCs) were counted in each well. The number of nuclei per cell and the size of TRAP-positive MNCs were measured using BZ-X analyzer (Keyence, Osaka, Japan).

### 2.9 Cell viability assay

Trypan blue staining was used to quantify viable cells. In brief, primary osteoblasts, RAW264.7 cells and mouse bone marrow cells were cultured with or without myonectin for 24 h. Next, the culture medium and cells were harvested, and a drop of the cell suspension was mixed with trypan blue solution. The ratio of each volume was 1:1. The total numbers of viable and nonviable cells were determined under a light microscope, and the data were expressed as the percentage of viable per total cells per well.

### 2.10 Oxygen consumption measurement

For measurement of the oxygen consumption rate (OCR), RAW264.7 cells, primary osteoblasts and mouse bone marrow cells were analyzed with an XF96 Extracellular Flux Analyzer with a Mito Stress kit (Seahorse Bioscience, North Billerica, MA, USA). RAW 264.7 cells were seeded in XF96 cell culture microplates (2.5×10^3^ cells/well) and cultured with 75 ng/ml RANKL for 4 days. Primary osteoblasts were seeded in XF96 cell culture microplates (3×10^3^ cells/well) and cultured for 3 days. Mouse bone marrow cells were seeded in XF96 cell culture microplates (5×10^3^ cells/well) and cultured with 50 ng/ml M-CSF for 3 days. Then, the bone marrow cells were cultured with 50 ng/ml M-CSF and 75 ng/ml RANKL for further 4 days. Culture medium was changed to Agilent Seahorse XF Base Medium supplemented with glucose (10 mM), sodium pyruvate (1 mM) and L-glutamine (2 mM) 1 h before measurement. The basal OCR and the OCR after injections of 1 μM oligomycin, 0.5 μM carbonyl cyanide 4-(trifluoromethoxy) phenylhydrazone (FCCP), and 0.5 μM rotenone/0.5 μM antimycin A for three measurement cycles at each step were analyzed.

### 2.11 Statistical analysis

Data are expressed as the mean ± the standard error of the mean (SEM). The results represent experiments performed at least independently 3 times. Statistical significance was evaluated using the Mann-Whitney *U* test for comparisons of two groups. One-way or two-way analysis of variance followed by the Tukey-Kramer test or Dunnett test was performed for multiple comparisons. The significance level was set at *P* < 0.05. All statistical analyses were performed using GraphPad PRISM 7.00 software.

## 3 Results

### 3.1 Effects of myonectin on mouse bone cells

**Figure 1:**
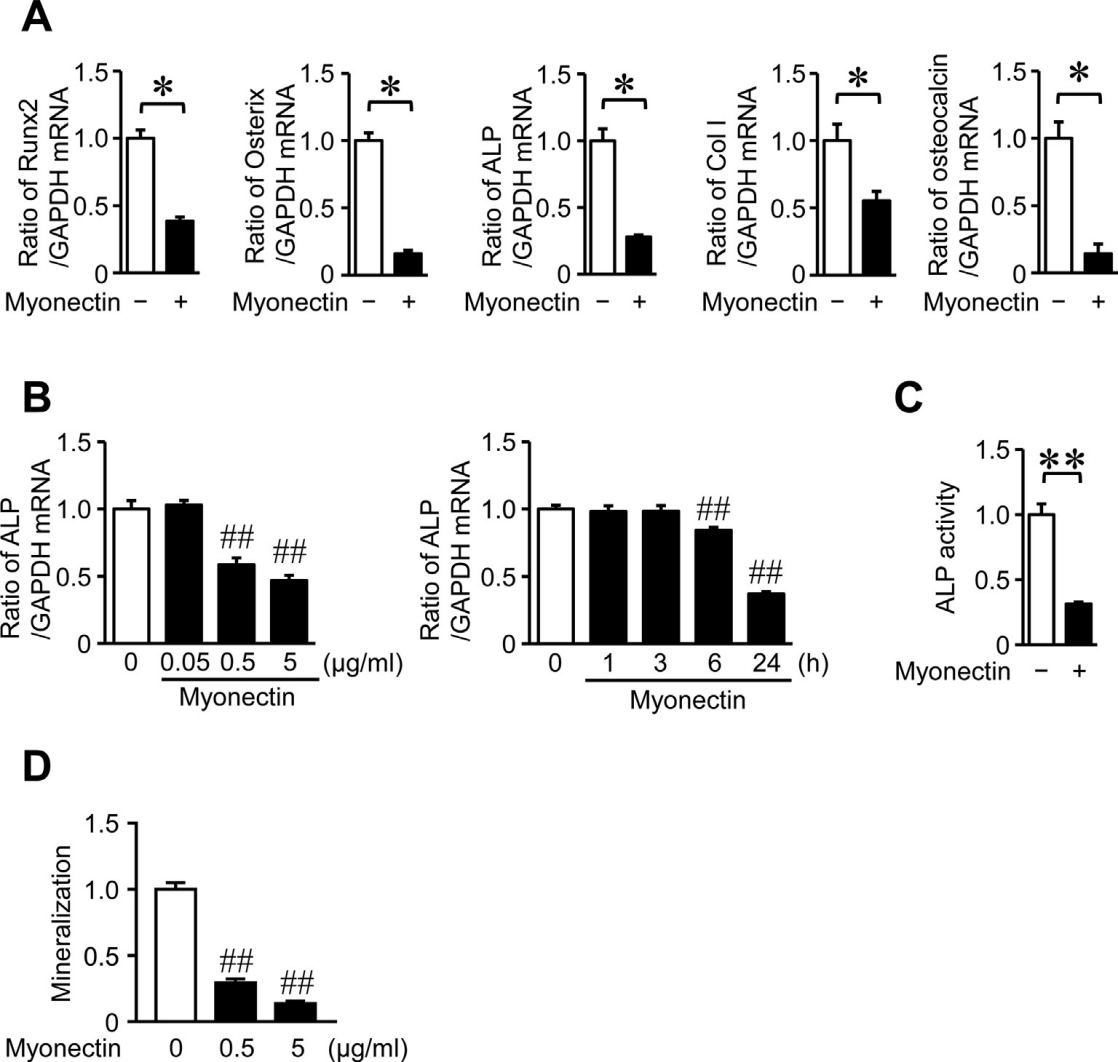
Effects of myonectin on the osteoblast differentiation. (A) Total RNA was extracted from mouse osteoblasts cultured with or without 5 μg/ml myonectin for 24 h, and real-time PCR analysis of Runx2, Osterix, ALP, type I collagen (Col1), osteocalcin or GAPDH was performed. Data represent the mean ± SEM of 4 experiments in each group. ∗*P* < 0.05 (Mann-Whitney *U* test). (B) Total RNA was extracted from mouse osteoblasts cultured with the indicated concentrations of myonectin for 24 h or 1 μg/ml myonectin for the indicated times, and real-time PCR analysis of ALP or GAPDH was performed. Data represent the mean ± SEM of 4 experiments in each group. ##*P* < 0.01 versus control (Dunnett test). (C) ALP activity was measured in confluent mouse osteoblasts cultured with or without 1 μg/ml myonectin for 24 h as described in Materials and Methods. Data represent mean ± SEM of 5 experiments in each group. ∗∗*P* < 0.01 (Mann-Whitney *U* test). (D) Mouse osteoblasts were cultured with 10 mM β-glycerophosphate in the presence or absence of 0.5 or 5 μg/ml myonectin for 3 weeks (n = 4 in each group). Mineralization was determined by Alizarin red staining as described in Materials and Methods. Data represents mean ± SEM of 4 experiments in each group. ##*P* < 0.01 versus control group (Dunnett test).

We examined the effects of myonectin on mouse osteoblastic cells and osteoclast formation *in vitro*. As shown in Figure 1A, myonectin significantly suppressed the mRNA levels of Runx2, Osterix, ALP, type I collagen and osteocalcin, osteogenic factors, in mouse primary osteoblasts. The effects of myonectin on the mRNA levels of ALP were dose-dependent and observed at 6 h and more than (Figure 1B). Moreover, myonectin significantly suppressed ALP activity and mineralization in mouse osteoblasts (Figures 1C, 1D). 1 and 5 μg/ml myonectin did not affect cell viability of mouse osteoblasts in trypan blue stain (Cell viability: Control, 93.4 ± 0.6%; 1 μg/ml myonectin, 92.3 ± 1.1%; 5 μg/ml myonectin, 89.8 ± 1.2%; n = 6 in each group).

**Figure 2:**
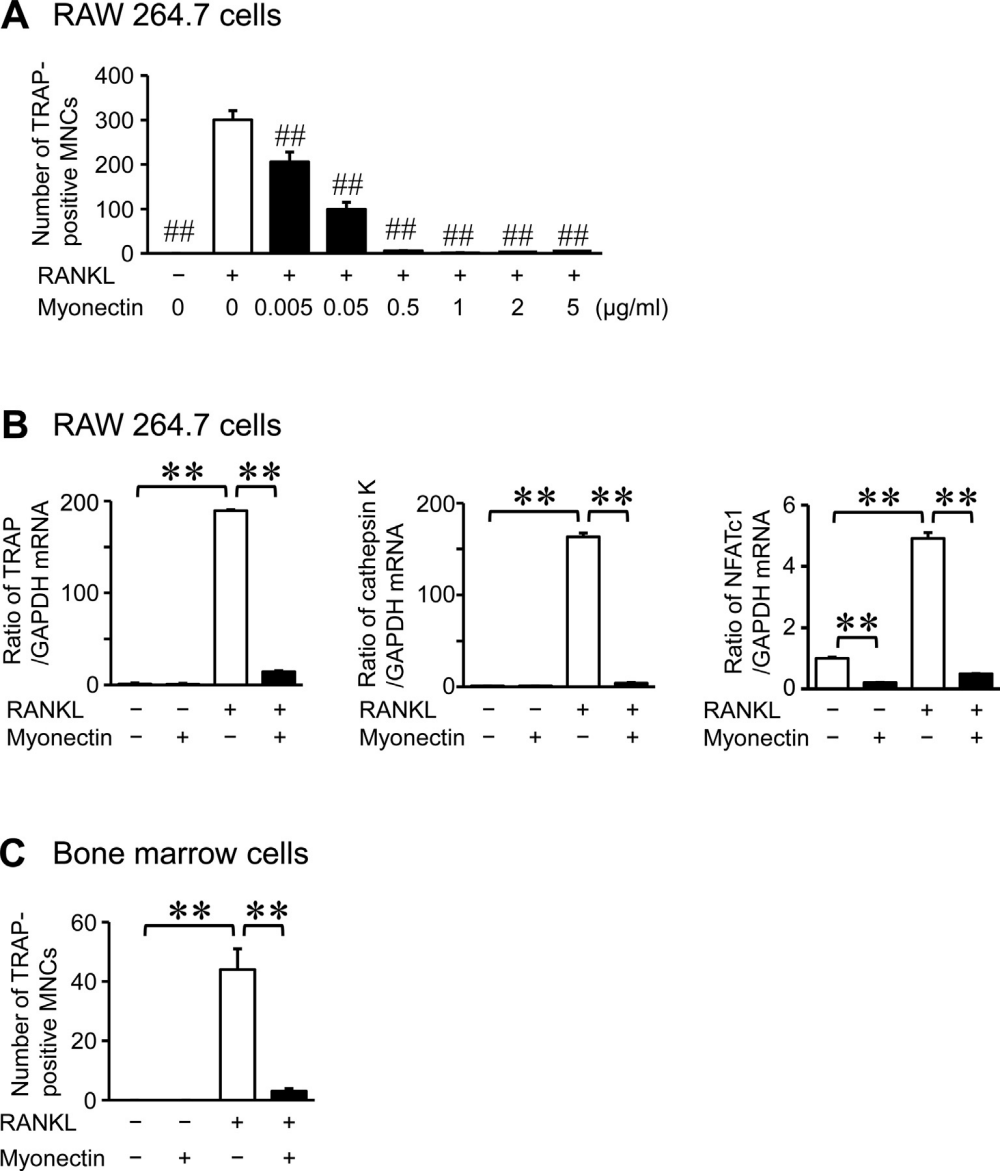
Effects of myonectin on osteoclast formation. (A) Osteoclast formation was induced by treatment with RANKL (75 ng/ml) in RAW264.7 cells in the presence or absence of the indicated concentrations of myonectin for 4 days. The cells were stained with TRAP staining, and the number of TRAP-positive multinucleated cells (MNCs) was counted in each well. The data represent the mean ± SEM of 4 experiments. (B) Total RNA was extracted from RAW264.7 cells cultured with or without 0.5 μg/ml myonectin in the presence or absence of RANKL (75 ng/ml) for 4 days, and real-time PCR analysis of TRAP, cathepsin K, NFATc1 or GAPDH was performed. Data represent the mean ± SEM of 4 experiments in each group. (C) Osteoclast formation was induced by treatment with M-CSF (50 ng/mL) and RANKL (75 ng/mL) in bone marrow cells in the presence or absence of 0.5 μg/ml myonectin for 4 days. The cells were stained with TRAP staining, and the number of TRAP-positive MNCs was counted in each well. The data represent the mean ± SEM of 4 experiments. ##*P* < 0.01 versus RANKL-treated group (A; Dunnett test). ∗∗*P* < 0.01 (B, C; Tukey-Kramer test).

**Figure 3:**
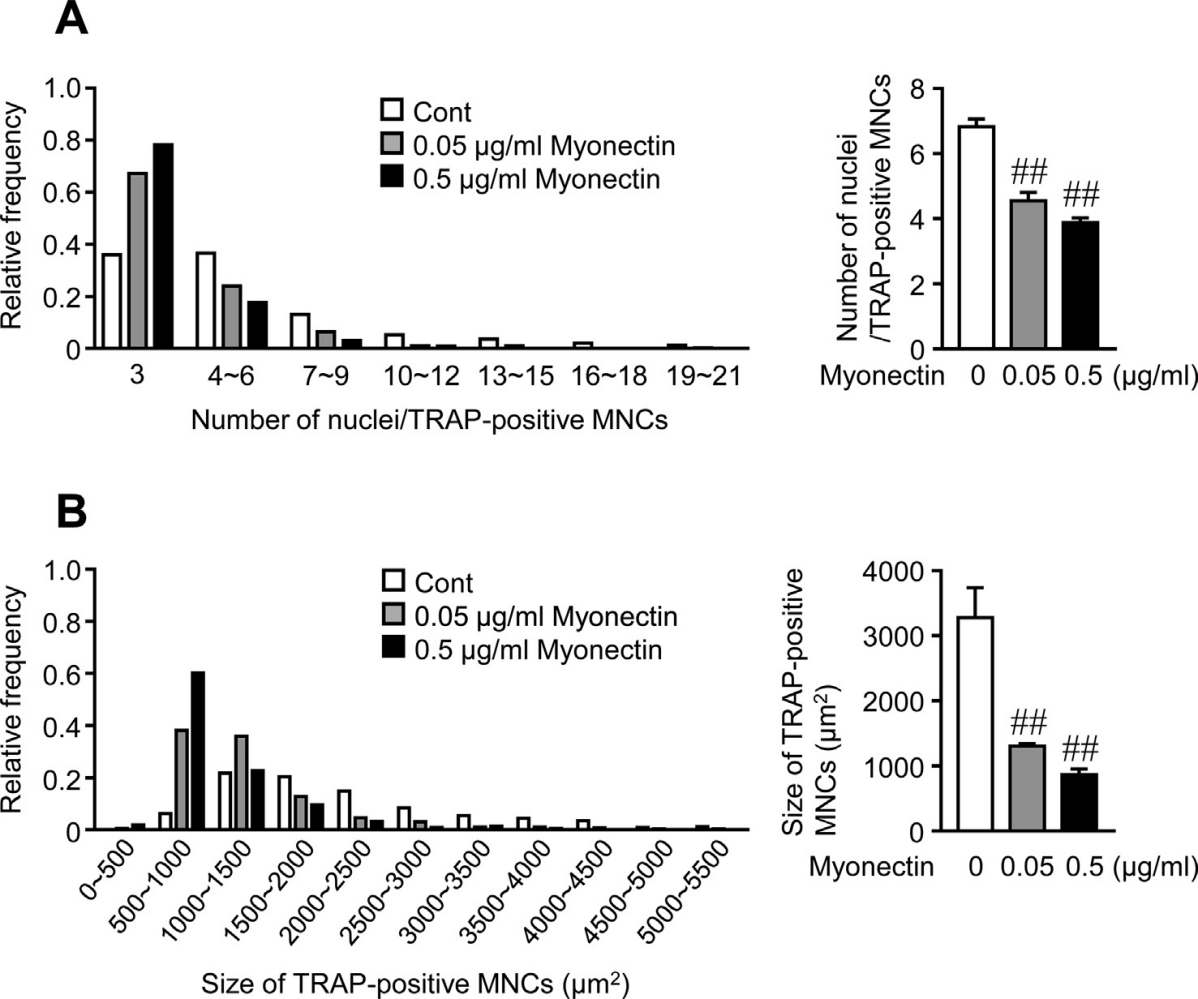
Effects of myonectin on osteoclast size and nuclear number. (A, B) Osteoclast formation was induced by treatment with RANKL (75 ng/ml) in RAW264.7 cells in the presence or absence of the indicated concentrations of myonectin for 4 days. The cells were stained with TRAP staining, and the number of nuclei per TRAP-positive multinucleated cells (MNCs) (A) and the size of TRAP-positive MNCs (B) were counted in each well. The data represent the relative frequency (upper left panel: n = 428, 391 and 221 cells in control, 0.05 μg/ml myonectin and 0.5 μg/ml myonectin groups, respectively; lower left panel: n = 380, 384 and 221 cells in control, 0.05 μg/ml myonectin and 0.5 μg/ml myonectin groups, respectively). The data represent the mean ± SEM of 4 experiments (right panels: n = 4 in each group). ##*P* < 0.01 versus control group (Dunnett test).

Next, we examined the effects of myonectin on osteoclast formation. Myonectin significantly suppressed osteoclast formation from mouse monocytic RAW264.7 cells in the presence of RANKL, and these effects were dose-dependent (Figure 2A). Moreover, myonectin suppressed the mRNA levels of TRAP, cathepsin K and nuclear factor of activated T cells (NFATc1) enhanced by RANKL in these cells (Figure 2B). In addition, myonectin significantly suppressed osteoclast formation from mouse bone marrow cells in the presence of M-CSF and RANKL (Figure 2C). Myonectin significantly suppressed the number of nuclei per cell and the size of TRAP-positive MNCs from RAW264.7 cells in the presence of RANKL (Figure 3A, B). 0.05 and 0.5 μg/ml myonectin did not affect cell viability of RAW264.7 and mouse bone marrow cells in trypan blue stain (Cell viability in RAW264.7 cells: Control, 96.3 ± 0.4%; 0.05 μg/ml myonectin, 96.3 ± 0.6%; 0.5 μg/ml myonectin, 95.6 ± 0.6%; n = 6 in each group. Cell viability in mouse bone marrow cells: Control, 93.0 ± 0.7%; 0.5 μg/ml myonectin, 94.6 ± 0.4%; n = 6 in each group).

### 3.2 Effects of myonectin on mitochondrial energy metabolism

**Figure 4:**
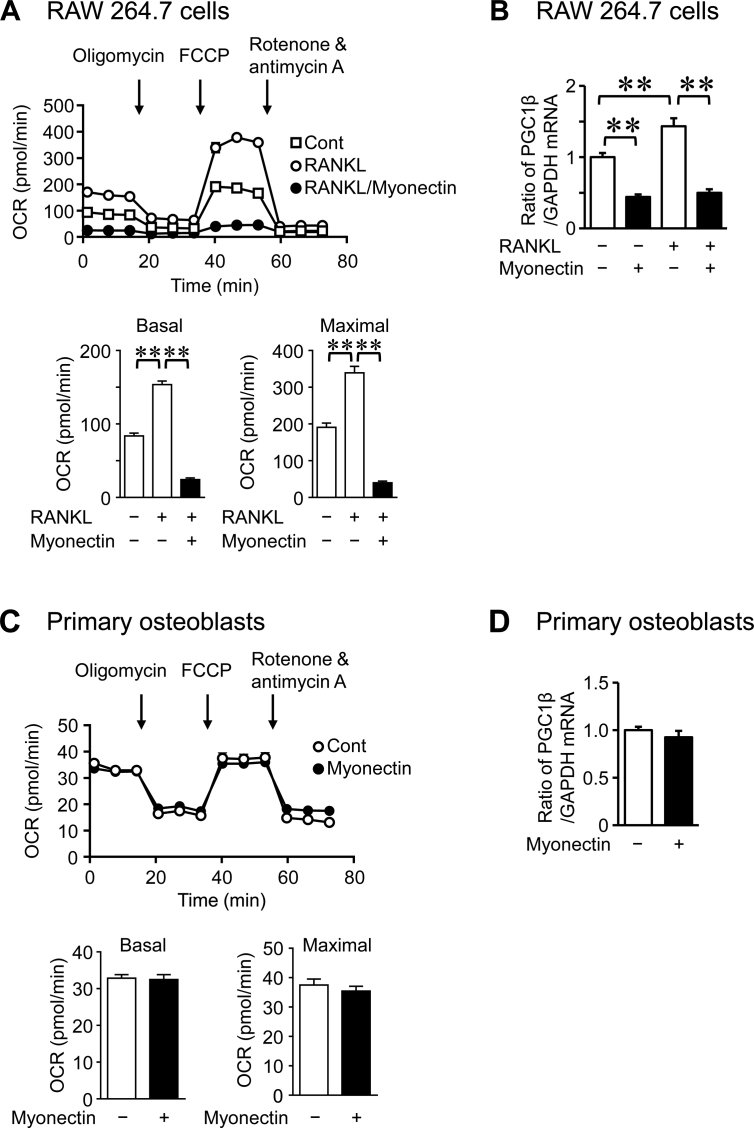
Effects of myonectin on mitochondrial energy metabolism in RAW264.7 cells and osteoblasts. (A) RAW264.7 cells were cultured with or without myonectin (0.5 μg/ml) in the presence or absence of RANKL (75 ng/ml) for 4 days. The OCR of cells was analyzed with an XF96 Extracellular Flux Analyzer. Basal OCR (before stimulation with oligomycin) and maximal OCR (after stimulation with FCCP) were measured. Data represent the mean ± SEM of 6 experiments in each group. ∗∗*P* < 0.01 (Tukey-Kramer test). (B) Total RNA was extracted from RAW264.7 cells cultured with or without myonectin (0.5 μg/ml) in the presence or absence of RANKL (75 ng/ml) for 4 days, and real-time PCR analysis of PGC1β or GAPDH was performed. Data represent the mean ± SEM of 4 experiments in each group. ∗∗*P* < 0.01 (Tukey-Kramer test). **(C)** Mouse osteoblasts were cultured with or without myonectin for 24 h. OCR values of cells were analyzed with an XF96 Extracellular Flux Analyzer. Basal OCR (before stimulation with oligomycin) and maximal OCR (after stimulation with FCCP) were measured. Data represent the mean ± SEM of 12 experiments in each group. (D) Total RNA was extracted from mouse primary osteoblasts cultured with or without myonectin (1 μg/ml) for 24 h, and real-time PCR analysis of PGC1β or GAPDH was performed. Data represent the mean ± SEM of 4 experiments in each group.

**Figure 5:**
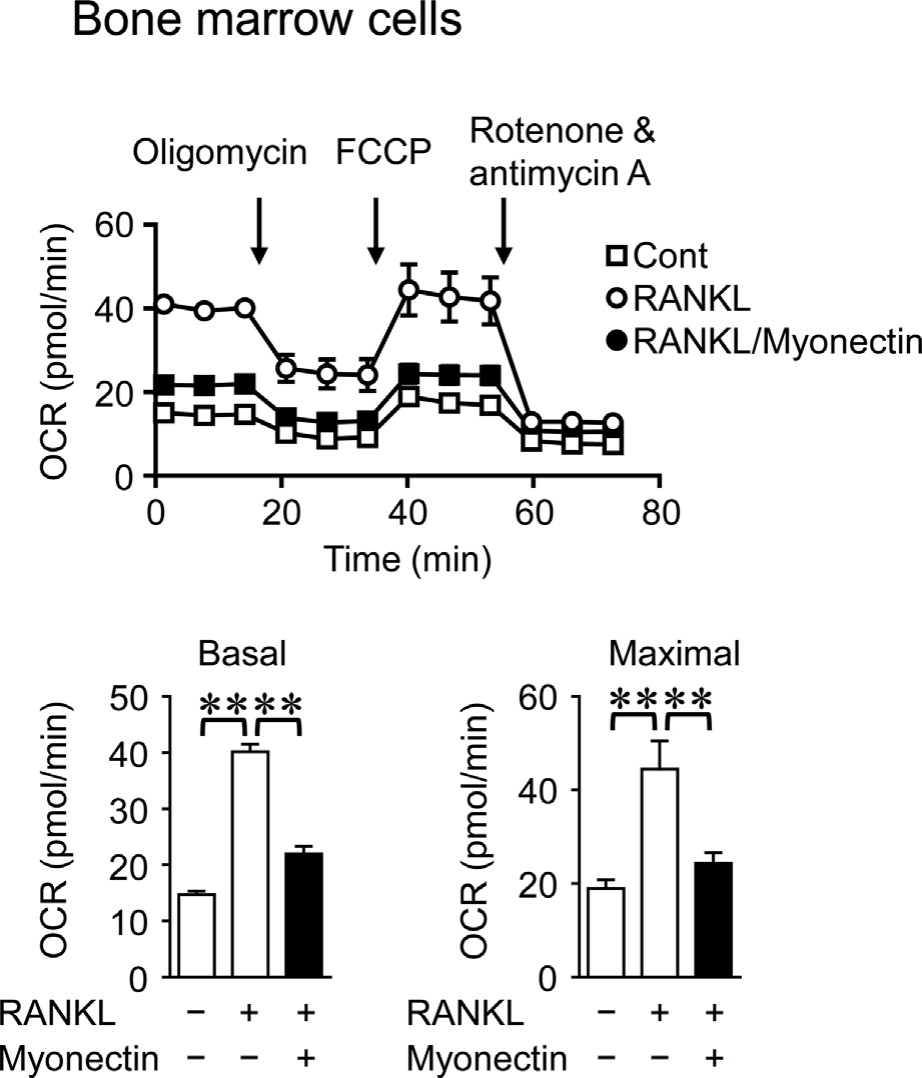
Effects of myonectin on mitochondrial energy metabolism in mouse bone marrow cells. Mouse bone marrow cells were cultured with M-CSF (50 ng/ml) for 3 days. Then, the cells were cultured with or without myonectin (0.5 μg/ml) in the presence or absence of RANKL (75 ng/ml) for further 4 days. The OCR of cells was analyzed with an XF96 Extracellular Flux Analyzer. Basal OCR (before stimulation with oligomycin) and maximal OCR (after stimulation with FCCP) were measured. Data represent the mean ± SEM of 6 experiments in each group. ∗∗*P* < 0.01 (Tukey-Kramer test).

Previous study indicates that the mitochondrial energy metabolism is changed during osteoclast differentiation in mice [17]. We therefore examined the effects of myonectin on mitochondrial biogenesis to investigate the mechanisms by which myonectin suppresses osteoblast and osteoclast differentiation in mouse cells. Peroxisome proliferator-activated receptor γ coactivator 1-β (PGC1β) is a key regulator of mitochondrial biogenesis [18]. As shown in Figures 4A and 4B, myonectin significantly suppressed OCR and PGC1β mRNA levels enhanced by RANKL in RAW264.7 cells. On the other hand, myonectin did not affect OCR and PGC1β mRNA levels in mouse osteoblasts (Figure 4C, D), although myonectin significantly suppressed OCR enhanced by M-CSF and RANKL in mouse bone marrow cells (Figure 5).

## 4 Discussion

Recent accumulating evidence suggests that various myokines influence bone in the pathophysiology of osteoporosis, such as immobilization, diabetes, glucocorticoid excess [12]. Myostatin, interleukin-6, and transforming growth factor-β negatively affect bone cells, although irisin, insulin-like growth factor-1, fibroblast growth factor-2 and follistatin might exert positive effects on bone [12]. In the present study, we first revealed that myonectin suppresses the differentiation and ALP activity in mouse osteoblasts. Moreover, it suppressed osteoclast differentiation from mouse bone marrow and RAW264.7 cells, although the effects of myonectin on bone resorbing activity of osteoclasts have still remained unknown in the present study. These findings suggest that myonectin might be involved in the interactions between muscle and bone in the pathophysiology of osteoporosis.

Several studies suggest that numerous factors affect bone cells by acting on mitochondrial function [19, 20]. Esen et al. revealed that an enhancement of aerobic glycolysis is related to an anabolic action of parathyroid hormone on bone in mice [19]. Zhang et al. reported that irisin stimulates osteoblast differentiation through an enhancement of aerobic glycolysis in mouse osteoblasts [20]. Although osteoclasts are rich in mitochondria for the high energy demands, mitochondrial biogenesis is crucial for osteoclast differentiation [17, 18]. In the present study, myonectin suppressed OCR and PGC1β expression in RAW264.7 cells, although myonectin did not affect OCR and PGC1β in mouse osteoblasts. These data suggest that myonectin inhibits osteoclast differentiation partly through mitochondrial biogenesis in mice, although the effects of myonectin on osteoblast differentiation and ALP activity seemed to be independently of mitochondrial function in mouse osteoblasts. This discrepancy of myonectin action on mitochondrial biogenesis between osteoblasts and osteoclasts seemed to be similar with our previous findings that muscle cell-derived extracellular vesicles suppress mitochondrial biogenesis in mouse osteoclast precursors, but not in mouse osteoblasts [21]. The studies by Seldin et al. indicate that myonectin suppresses the autophagy through phosphoinositide 3-kinase (PI3K)/Akt/mammalian target of rapamycin (mTOR) pathway in liver [1, 7]. Otaka et al. reported that myonectin attenuates cardiac myocyte apoptosis and macrophage inflammatory response to lipopolysaccharide through sphingosine-1-phosphate-induced cAMP/Akt pathway in mouse cells [11]. The detailed mechanisms of myonectin action and its receptor/binding protein have remained unknown.

The risk of osteoporotic fractures is increased in patients with diabetes, and a decrease in bone turnover is related to the pathophysiology of diabetic osteoporosis [22]. An elevation in serum myonectin level is associated with diabetic state and the development of diabetes in humans and animals [6, 9, 23, 24]. Our present study indicated that myonectin suppresses both osteoblast function and osteoclast differentiation in mouse cells, which might lead to low turnover of bone metabolism *in vivo*. These findings are compatible with low turnover bone state in diabetic state. We can speculate that myonectin might contribute the pathophysiology of diabetic osteoporosis by suppressing both osteoblastic bone formation and osteoclastic bone resorption. Further studies are necessary to investigate the roles of myonectin *in vivo* study.

In conclusion, we first demonstrated that myonectin suppresses osteoblastic differentiation and ALP activity of mouse osteoblastic cells as well as osteoclast differentiation from mouse bone marrow and RAW264.7 cells. An inhibition of mitochondrial biogenesis might be related to the mechanism by which myonectin suppresses osteoclast differentiation.

## Declarations

### Author contribution statement

H. Kaji: Conceived and designed the experiments; Analyzed and interpreted the data; Contributed reagents, materials, analysis tools or data; Wrote the paper.

M. Kawaguchi: Performed the experiments; Analyzed and interpreted the data; Contributed reagents, materials, analysis tools or data; Wrote the paper.

N. Kawao: Performed the experiments; Analyzed and interpreted the data; Contributed reagents, materials, analysis tools or data.

Y. Takafuji and M. Ishida: Performed the experiments.

### Funding statement

N. Kawao was supported by a Grant-in-Aid for Scientific Research (C: 19K07310) and H. Kaji was supported by a Grant-in-Aid for Scientific Research on Innovative Areas (grant number 15H05935, "Living in Space") from the 10.13039/501100001700Ministry of Education, Culture, Sports, Science and Technology, Japan.

### Competing interest statement

The authors declare no conflict of interest.

### Additional information

No additional information is available for this paper.

## Appendix A Supplementary data

TableS1.docx TableS1.docx
