# Supplementary material for: Myonectin inhibits the differentiation of osteoblasts and osteoclasts in mouse cells
Source: Heliyon. 2020 May 15;6(5):e03967. doi: 10.1016/j.heliyon.2020.e03967 (PMC7266783; doi:10.1016/j.heliyon.2020.e03967)
Supplement: TableS1.docx [file mmc1.docx]

**Table S1** Primers used for real-time PCR experiments.

| Gene |  | Primer sequence |
| --- | --- | --- |
| Runx2 | Forward  Reverse | 5’-AAATGCCTCCGCTGTTATGAA-3’  5’-GCTCCGGCCCACAAATCT-3’ |
| Osterix | Forward  Reverse | 5’-AGCGACCACTTGAGCAAACAT-3’  5’-GCGGCTGATTGGCTTCTTCT-3’ |
| ALP | Forward  Reverse | 5’-ATCTTTGGTCTGGCTCCCATG-3’  5’-TTTCCCGTTCACCGTCCAC-3’ |
| Col I | Forward  Reverse | 5’-AACCCTGCCCGCACATG-3’  5’-CAGACGGCTGAGTAGGGAACA-3’ |
| Osteocalcin | Forward  Reverse | 5’-CCTGAGTCTGACAAAGCCTTCA-3’  5’-GCCGGAGTCTGTTCACTACCTT-3’ |
| TRAP | Forward  Reverse | 5’-CAGCTGTCCTGGCTCAAAA-3’  5’-ACATAGCCCACACCGTTCTC-3’ |
| Cathepsin K | Forward  Reverse | 5’-GAGGGCCAACTCAAGAAGAA-3’  5’-GCCGTGGCGTTATACATACA-3’ |
| NFATc1 | Forward | 5’-CAAGTCTCACCACAGGGCTCACTA-3’ |
|  | Reverse | 5’-GCGTGAGAGGTTCATTCTCCAAGT-3’ |
| PGC1β | Forward | 5’-CCTCATGCTGGCCTTGTCA-3’ |
|  | Reverse | 5’-TGGCTTGTATGGAGGTGTGG-3’ |
| GAPDH | Forward | 5’-AGGTCGGTGTGAACGGATTTG-3’ |
|  | Reverse | 5’-GGGGTCGTTGATGGCAACA-3’ |

ALP, alkaline phosphatase; Col I, type I collagen; TRAP, tartrate-resistant acid phosphatase; NFATc1, nuclear factor of activated T cells; PGC1β, peroxisome proliferator-activated receptor gamma coactivator 1-β; GAPDH, glyceraldehyde-3-phosphate dehydrogenase.
